# Supplementary material for: Severe childhood anemia and emergency blood transfusion in Gadarif Hospital, eastern Sudan
Source: PLoS One. 2019 Dec 3;14(12):e0225731. doi: 10.1371/journal.pone.0225731 (PMC6890167; doi:10.1371/journal.pone.0225731)
Supplement: Attachment — Submitted filename: Figure 1_PONE-D-19-15934.docx [file pone.0225731.s002.docx]

Figure 1.(line 154-166): The flow chart: Consider revising this flow chart, such as below (only a guide);
