# Supplementary material for: Severe childhood anemia and emergency blood transfusion in Gadarif Hospital, eastern Sudan
Source: PLoS One. 2019 Dec 3;14(12):e0225731. doi: 10.1371/journal.pone.0225731 (PMC6890167; doi:10.1371/journal.pone.0225731)
Supplement: Attachment — Submitted filename: Suggested table .docx [file pone.0225731.s003.docx]

Patient’s characteristics

|  | **n.** | **Age**  Mean (± SD) | **Hb**  Mean (± SD) |
| --- | --- | --- | --- |
| **Admitted** | 1800 |  |  |
| **Non anemic** | 1287 (71.5) |  |  |
| **Anemic** | 513 (28.5) |  |  |
| Mild – moderate* | 372 (20.6) |  |  |
| Severe* | 141 (7.8) |  |  |
| **Received transfusion** | 300 (13.6) |  |  |
| Age < 5 years | 151 (50.3) |  |  |
| Hb < 4.0 g/dL | 66 (25.7) |  |  |
| Hb ≥ 4 - ≤ 7 g/dL | 204 (74.3) |  |  |
| Improved | 285 (95.0) |  |  |
| Discharged against medical advice | 9 (3.0) |  |  |
| Died | 6 (2.0) |  |  |

* see remarks for the classification criteria
