# Supplementary material for: Severe childhood anemia and emergency blood transfusion in Gadarif Hospital, eastern Sudan
Source: PLoS One. 2019 Dec 3;14(12):e0225731. doi: 10.1371/journal.pone.0225731 (PMC6890167; doi:10.1371/journal.pone.0225731)
Supplement: Attachment — Submitted filename: renamed_bb956.docx [file pone.0225731.s004.docx]

We would like to thank the editor and reviewers for their valuable comments on this manuscript. We greatly appreciate your time dealing with the manuscript and assessing the review comments, which enabled us to greatly improve the quality of our manuscript. Here is a point-by-point response to the reviewers’ comments and concerns

5. Review Comments to the Author
Summary: This was a cross sectional study conducted at a tertiary hospital in Eastern Sudan, in which the investigators attempted to determine the clinical use of blood among children receiving urgent transfusion. They found that approximately 56.5% (300/531) of children with anemia presenting to the emergency unit were transfused. Sickle cell disease (43%), acute bleeding (19.3%) and malaria (16.7%) were the leading diagnostic categories for which urgent blood transfusions were given.
Overall impression
The authors conducted a descriptive study, but methods of which lack details. There is a major concern for selection bias, since hemoglobin electrophoresis was only done among some patients, and yet sickle cell disease turned out to be the leading diagnostic category for transfusion. The data analysis seems incomplete (for the type of study design), since they could not even establish the factors associated with the main outcome variables. Consequently, the claims/answers to the current study are not supported by credible results. The authors make several speculations, such as about HIV, Schistosomiasis, and Sickle cell disease with no supporting data. Similarly, the results of some stated objectives are either not available or cancelled; probably for tests that were never performed. Results presentation and discussion deserve major revisions.
Specific issues, in details

Comment
Major issues
a) Introduction:
1. The objective(s) of the study, as stated in line 92-96, are not clear. Please revise, and clarify on the following:
i) Was the aim to ‘assess the rate’ of anemic children who….or to the ‘determine the proportion’ of anemic children who receive transfusion….?
ii) Did the authors examine (the indication, patterns and outcomes…), or describe?

Response
Objectives were revised, the word “assess” was replaced with “determine" and “describe” instead of “examine” in the specified lines as suggested. The word pattern was deleted from the objectives because it was not investigated. Please see lines 94- 97.

Comment
b) Methods:
1. The authors need to clarify on the type of study design (line 98). Was this a cross-sectional study?

Response

Cross-sectional study was added. Please see the method line 100.

Comment
2. Generally, the methods lack details. By providing details of the scientific methods used in a clinical study, the researcher assures the global community that – another person, using the same tools may reproduce similar findings. Please explain and clarify on the following:
i) Samples of urine and stool were taken off (line 113); Provide details of the specific tests that were performed, and the machines used.

Response

Urine and stool analysis was conducted for schistosomal infection as described before. This has been inserted in the method. Please see the methods line 116.

Comment
ii) Line 114-115, mentions that blood was examined for malaria. Please provide details; which films: thick films, thin films, or both? Which stains were used? Who read them?

Response

Blood was examined for malaria using thick and thin blood films which were stained with 10% Giemsa and read by expert microscopist. This point has been inserted in the method. Please see it in lines 117-118.

Comment

iii) The diagnosis of anemia is very crucial in this study (refer to line 116). How was hemoglobin determined? Please provide details (e.g which machine was used?)

Response

Hemoglobin estimation was done as part of the complete hemogram via automated blood-cell analyzer machine (Sysmex Hematology Analyzer; Sysmex, Kakogawa, Japan). This point has been inserted in the method as suggested. Please see lines 118-120.

Comment
iv) Hemoglobin electrophoresis (refer to line 116). Provide details of the machine and techniques used.

v) Hemoglobin electrophoresis (refer to line 116). The authors say this was done “if required in some cases”! This is a major concern for selection bias, if such a test that confirms sickle cell status was only performed among some patients. Remarkably sickle cell disease turned out to be the leading diagnostic category for transfusion, yet some children were NOT tested! Please explain.

Response

The test was performed for those who were suspected clinically to have sickle cell disease; the test was not done to already diagnosed patients, we depended on the recorded results from past tests. Haemoglobin genotype using the usual electrophoretic method (electrophoretic equipment model MUPID-EXU, Japan). Please see lines 127-131.

Comment
vi) In line 123, the authors state that transfusions were given for longer than 4 hours. However, transfusions are generally given for a period 2-4hrs. The practice of transfusion for longer than 4hrs is not safe and increases the risk for bacterial contamination. Is this the standard practice at Gadarif hospital? Please Explain.

Response

Yes it is correct that , transfusions are generally given for a period 2-4hrs. It was typo error (we mean for not longer than) it has been corrected. Please see the text line 138

Comment
vii) Similarly, explain how the diagnosis /suspicion of pulmonary edema was made. There is a risk for irrational use of frusemide.

Response

Pulmonary edema was clinically determined if the patient had shortness of breathing, increased respiratory rate with coarse crackles at the lung bases and radiologically as defined by the presence of Kerley B lines in the anteroposterior chest viewwas explained in the text. This point has been inserted in the method lines 140-143.

Comment
viii) The text in line 126 is confusing. Do you mean “the time taken from order, to actual transfusion”? This may not be referred to as ‘time of blood transfusion’. Please modify.

Response

The sentence was modified to “The time taken from order of blood to actual transfusion” Please see it now line 151.

Comment
ix) There are many other details missing, that should be mentioned such as:
x) How was the diagnosis of acute malnutrition made? Provide details.

Response

Details of diagnosis of malnutrition were added in the methods “Severe acute malnutrition was diagnosed following the WHO guidelines for malnutrition. A child was considered as severe acute malnutrition if weight-for-height z score was <-2 SD for age and sex or presence of bilateral lower limb edema”. Please see it lines 143-146.

Comment
xi) How was the diagnosis of visceral leishmaniasis made? Provide details.

Response

Details of diagnosis of leishmaniasis were added “The diagnosis of visceral leishmaniasis was confirmed by the visualization of the amastigote form of the parasite by microscopic examination of aspirates from or bone marrow using Giemsa-stain”. Lines 146-148.

Comment
xii) How was the diagnosis of sepsis made?

Response

Diagnosis of sepsis was explained in the text” The definition of sepsis is considered as “life threatening organ dysfunction caused by a dysregulated host response to infection[25]. Lines 149-150.

Comment
3. Statistical analysis:
i) Generally, additional statistical analysis needs to be done, such as univariate and bivariable analysis - to establish factors associated with the main outcomes such as survival, etc.

Response

We agreed that univariate and bivariable analysis is needed. However, there are few numbers “ 9 children discharged against medical advice and 6 children died” in the outcome. If we tried to conduct bivariable analysis in this case the model will be distorted with a wide range of confidence intervals. Hence this is the reason behind that we did not conduct it.

Comment

ii) Line 145: were all the numerical data symmetrical, to justify the use of Mean (SD)? If not, use median for asymmetrical data. Please check and revise.

Response

Yes Continuous data were checked for normality using Shapiro-Wilk test and they were presented as mean (Standard Deviation – SD) if they were normally distributed or median [interquartile range – IQR] if they were not normally distributed. This point has been inserted in the statistics. Please see lines 173-175.

Comment

4. For this study, the authors need to specify their operational definitions. For example;
i) What defined malaria?
ii) What defined sickle cell disease?
iii) What defined acute malnutrition?
iv) What defined acute bleeding?..etc.
to add the other definitions

Response

All these have been defined as suggested. Please see the methods and mentioned above.

Comment
c) Results:
1. Generally when presenting results, Table 1; which is the table of baseline characteristics comes up-front. These characteristics may include; gender, age, duration of illness, relationship with primary caregiver, occupation of caregiver, ethnic group/tribe etc. This table is missing.

Response

The suggested table has been inserted. Thank you very much indeed as you draw its outlines.

Comment
2. Present the results systematically; objective by objective.

Systematic presentations of the results
3. Results of some stated objectives are missing, such as; ‘pattern’ and ‘time taken from order, to actual transfusion’. Provide them.

Results of pattern, time taken from order to actual transfusion.

Response

The pattern was not investigated and it has been deleted from the objectives. The others have been put in order as suggested.

Comment
4. Samples of urine and stool were taken off. Provide the results of these tests.

Response

It has been added” There was no case of intestinal or urinary [schistosomiasis](https://www.ncbi.nlm.nih.gov/pubmed/30309089)” Please see line 237

Comment
5. Hemoglobin electrophoresis was done. Provide detailed results, including the proportion with SS, AS etc.

Response

The details have been inserted in the results. Please see lines 236.
Comment

6. Figure 1(line 154-166): The flow chart:
Consider revising this flow chart, such as below (only a guide);…Chart is attached, separate file,

Response

The suggested revised chart has been added. Thank very much indeed for this suggestion.
Comment
7. The authors should account for each study participant. For example, 141/300 transfused children were categorized as severe anemia. What was the diagnosis among the 159 with Hb>5 but got transfused? Please explain and modify.

Response

Yes the details of blood transfusion in this group as” Of 204 children who had hemoglobin levels of 4–7 g/dl and were transfused; 110 (53.9%) had heart failure, 38 (18.6%) had < 20% of red blood cells parasitized by malaria parasites, 32 (15.7%) had continuing bleeding, 9(4.4%) had combined/ others reasons, 8(3.9%) had dehydration and 7(3.4%) had impaired level of consciousness” Please see lines 240-244.

Comment
8. These 159, deserve a thorough discussion (in the discussion section).

Add to discussion Hb>5 and transfused

Response

Please see above

Comment
9. This account above is important, because the description offered in line, 170 -173; does not seem to tie-up with what we normally see clinically: That 91% had pallor, 87% with fever and 64% with breathlessness, yet only 141 (47%) were diagnosed as severe anemia. Please clarify.

To explain why only 47% had severe anemia where 91% had pallor & 87% had fever

Response

Yes we agreed it is a valid point. The explanation for this point is that symptoms might not be a useful indicator for anemia. This point has been inserted in the discussion. Please see line 302-305.
Comment

10. Line 181: Figure 2: This is a duplication of presentation of findings. Generally it is not necessary to show both the text (177-180) and figure. You choose either.

Figure 2 to be deleted or otherwise choose the figure and sentence below

If choose text, then one writes...’the diagnosis associated with order of blood transfusion is shown in figure 2, below:’

Response

Oaky the text was removed and the figure is retained.

Comment
11. Line 189: For the five that developed adverse events, provide more details. E.g. After stopping, what happened to them? Full recovery? Where these 5 among the the 6 deaths? or the 9 that discharged self..

Response

The details have been inserted. Please see 248-250.

12. Fully account for each participant; such as in # c(11), and c(6) - c(8) above.

Response

Please see above

Comment
13. Line 190: The phrase; “A total of 285 children improved…” is left hanging. So what factors were associated with recovery? This entire paragraph (190-194) does not make sense, without additional statistical analysis, as pointed out in c(3) above. Please revise.
factors associated with recovery needed?????

Response

We agreed that there should be more analysis on the factors and their outcomes. As we mentioned above 285 out three hundreds improved. This left only 15 (died and discharged against medical advice) which is small number to perform bivariate analysis

Comment
d) Discussion:
1. What is the burden of Sickle cell disease in Sudan? In line 236, they mention one in 123 children. Do the authors believe this figure/prevalence? ….especially in light of their current finding that show; 129/300 (43%) of transfused children in that same setting have sickle cell disease? Please explain, and discuss.

Response

The (Misseriya tribes) ethnicity and the consanguineous marriages were the main explanations of the high prevalence (24.9%) of carriers of HbS allele (HbAS) in certain areas of Sudan. This point has been added to the discussion as suggested. Please see lines 308-310.

Comment
2. This section is generally poorly written (flow), with lots of repetitions. Suggested flow may include the following areas (paragraphs):
i) First paragraph – what was the aim of the study/ what was your question?
ii) What did you find (the answer to your question)
iii) A brief explanation of the findings, in comparison/contrast with other studies.
iv) What is new in the current study?
v) The strength of your study.
vi) Limitations
vii) Conclusions /or and recommendations.

Response

All these points have been addressed.
e) Conclusion:
Generally, the conclusion shall need to be revised, once most of the above revisions are finalized, especially those related to additional statistical analysis.

Response

All these have been addressed in the discussion as suggested.
Minor issues
Comment
a) Abstract:
1. Were all the 1,800 children evaluated for anemia? Please clarify.

Response

Yes were assessed for anemia. This point has been inserted in the abstract as suggested.

Comment
2. The text in line, 46-47 is confusion. “The diagnoses associated with the order for anemia…” Do clinicians order for anemia? Consider revising text, such as; “The diagnoses associated with the order for blood transfusion were…..” Do the same elsewhere, such as line 177.

Response
The sentence now read (diagnoses associated with the “severity” instead of “order” of anemia). It has been corrected throughout the paper as suggested

Comment
b) Introduction:
1. The text in line 72-73 lack clarity. Consider revising text, such as; ‘Childhood anemia is a major cause of morbidity and mortality globally.

Response
The sentence was rephrased

Comment
c) Methods:
1. Line 99, add ‘city’ to ‘Gadarif’ so that text reads; Gadarif city is 400km…..

Response

The word city is added now

Comment
2. Sample size estimation: Specify the formula used; such as Kish, Leslie for cross-sectional studies.

Response
Formula specification has been inserted as suggested.

Comment
d) Results,
1. Line 170: The authors need to use terminologies correctly and in context. By ‘breathlessness’ do the authors mean, respiratory distress or difficulty in breathing? Please clarify.

Response

Breathlessness explained as difficulty in breathing.

Comment
2. Similarly, Line 171: Palpitation is a rare symptom among children, especially in <5 years who often don't report symptoms. Remember 151 (50.3%) were <5 years! In addition, in table 1; palpitation is grouped as a sign! This is confusing. Please revise.

Response

I agreed it is confusing “ palpitation” has been deleted from the text and the table.

Commet
3. Line 184: revise the phrase, such as; Median time, from order to time of blood issue (or better start of transfusion) – as appropriate.

Response

The sentence was rephrased as: The median (interquartile range) request-to-issue time for blood was 6 (3−12) hours.

Comment
4. Line 186: For fever, generally we consider an increment of >1°C, from baseline. Was this the case? Please clarify.

Response

The fever was defined.

Comment
5. Line 187 (Adverse events are very important): Better specify; of the 5, how many got fever, and how many got urticarial rash?
Response

The details have been inserted as all of them had fever and urticarial rash. Please see lines 247-250.

Comment
e) Discussion:
1. Line 201: The authors state that, “the prevalence of anemia in our study was slightly higher than…”. Why? Please discuss. But, first clarify on how the 531(diagnosed with anemia) were got from the 1,800...to exclude selection bias. In other words, were all the 1,800 admitted children evaluated for anemia? Please clarify.

Response

This was clarified in the results section, please see above.

Comment
2. The phrase in line 215 conveys no meaning, especially with regard to what is put in parenthesis or (....). Please revise.

Response

Sentence rephrased

Comment
3. Line 216-217, about schistosomiasis: Are you sure? Please provide results of stool and urine, showing how many did not have. These results are missing.

Response

The sentence is rephrased as “Shistosomal infection was reported as the main cause of severe anemia of children….” This fact was cited from a previous study and not as a finding of this study.

Comment
4. Line 218-219; how did you know? When HIV was not tested. No mention of HIV testing in the methods or results. This should be one of the limitation of the study

Response

This part was deleted as you can see. The HIV was not tested, this has been inserted in the limitation of the study. Please see lines 341.

Comment
5. Line 252-253: This phrase is not accurate; 0.4% in Kiguli et al study is not comparable to 1.6%! Please revise or explain.

Response
The sentence was rephrased as follows: The “probable” blood transfusion reaction in our study was higher than that reported by [Kiguli](https://www.ncbi.nlm.nih.gov/pubmed/?term=Kiguli%20S%5BAuthor%5D&cauthor=true&cauthor_uid=25640706) et al
f) Other comments: None.
Reviewer #2: PLONE-D-19-15943
Title
Severe childhood anemia and emergency blood transfusion in Gadarif Hospital, eastern Sudan
GENERAL COMMENTS
In this study the Authors assessed the prevalence of anemia in children admitted to their hospital in Sudan with an indication for blood transfusion through an eight months period. We subsequently examined a series of pre- and post- transfusion parameters (underlying disease, symptoms, signs, patients’ outcome and others).
The study, as the first performed in Sudan in this field, appears original and useful to better understand the real prevalence of anemia, its causes, the indications to blood transfusion, the transfusion behavior in a pediatric emergency department and the patients’ outcome.
However, I have some remarks and suggestions for changes.
Comment

First of all, I think that a clear statement (with an appropriate reference) must be included with respect to the definition of anemia used by the Authors, because this can affect the results.
According to WHO, a child between 6 and 59 months is defined as “anemic” when the hemoglobin level is below 11.5 g/dl (“severely anemic” below 7 g/dl). Between 5 and 11 years “anemia” is present with Hb below 11.0 g/dl; between 12 and 14 years below 12.0 g/dl and from 15 years on below 13.0 g/dl. Except for the first age class, the definition of “severe anemia” is restricted to patients with hemoglobin level below 8.0 g/dl.
See: World Health Organization. Haemoglobin concentrations for the diagnosis of anaemia and assessment of severity. WHO; 2011.
Has to define all conditions in a paragraph titled operational definitions

Response

Yes thank you very much. The provided definition for anemia has been inserted as well the reference provided. Please see lines 121-126.

Comment
In the Results section of the abstract (line 42) the sentence was: “…513 (28.5%) were anemic (hemoglobin < 11 g/dl) and 141 (7.8%) had severe anemia” (hemoglobin level < 5 g/dl).” Please adjust this point.
Response

Okay thank you very much for your suggestion. The point has been adjusted.

Comment
In the statistical section the Authors declare that data are presented as mean (with standard deviation), but in line 184-185 they cite a “median (interquartile range)”. In line 194 the haemoglobin level should be expressed as median [IQR], especially since the word in brackets is “range”. Median and IQR are also cited in lines 242 and 244.

Clarification of mean interquartile usage in an expression of mean SD
In my opinion the sentence in statistical section should be “Data are presented as mean (± Standard Deviation – SD) or median [interquartile range – IQR]

Response

Yes we agreed and this has been adjusted all over the paper.

Comment

In the same sections the Authors should be described the methodology used to calculate the sample size (see line 132 – 135)
Response

Yes the sample size has been clarified. Please see lines 159-160
OTHER SPECIFIC COMMENTS
Introduction, Line 72 – 88
I would suggest a complete revision of the text, to make it more concise. The contents are appropriate, but there is a series of repetitions and redundancies.
Response

Repetitions and redundancies removed
Comment

Line 85
The reference n.12 (“Guidelines for informing about clinical decisions on transfusion associated with hemoglobin levels” ) doesn’t correspond to the WHO’s guidelines. In my opinion it could be better the reference n.20.

Response

Yes we agreed and it has been replaced by the suggested reference.
Reference 20 considered to replace reference 12
Line 123
Furosemide instead of frusemide
Response

Yes corrected as suggested
Comment

Line 135
A difference of 5% of what? Please specify.
Response

It is the precision (statistical term), it has been corrected.

Comment
Line 148 (Patients’ characteristics)
It is the only subheading in the “Results” section: the Authors may choose to erase it, to add other subheadings, if deemed appropriate.
Response

The subheading was deleted as suggested.
Comment

Line 151
The question is always the same: is the definition of severe anemia arbitrarily chosen or reference-based? It is sufficient to declare it.

Response
The definition of anemia is included as suggested

Comment
Figure 1. and lines 167-169
I suggest a table instead of a flowchart with the subsequent descriptive text.
The table is in the attachment named as “Suggested Table”
Response

The suggested table added

Comment
I would appreciate a bar chart with hemoglobin level classes at admission (e.g. < 4 / 4 - <5 / 5 - < 6 and so on) in the x axis and the prevalence in the y axis.
A bar chart to be added

Response

Oaky the requested figure has been inserted, please see figure 2.

Comment
Line 194
See “statistic section” in the General comments

Response
Statistics revised
Comment

Discussion
Line 202
I suggest to add the following reference: Kassebaum NJ, Jasrasaria R, Naghavi M, Wulf SK, Johns N, et al. A systematic analysis of global anemia burden from 1990 to 2010. Blood 2014(123):615–24. <https://doi.org/10.1182/blood-2013-06-508325>
Response

The reference is added
Comment

Line 251
I suggest to add also the following reference and include a short discussion on its different results respect to the articles in reference 2 and 11. Maitland K, Kiguli S, Olupot-Olupot P, Engoru C, Mallewa M, Saramago Goncalves P, et al. Immediate Transfusion in African Children with Uncomplicated Severe Anemia. New England Journal of Medicine. 2019 Aug 1;381(5):407–19.
Response

The reference is added, please see line
Comment

Conclusion
“Future reach on anemia is required” The phrase is not clear: please reword.
Response

It has been changed

Comment
Lastly, it could be interesting to know the level of blood transfusion appropriateness. Are the WHO guidelines always followed? Did the Authors document cases of undertransfusion or non-transfusion in patients in need for homologous blood supply?

To add the information of adherence to WHO guidelines on blood transfusion.

Response

 It is a valid point and thank you very much. However we did not think of it in our objectives. Perhaps we will include it in our future research.

**Regards**
