# Supplementary material for: Severe childhood anemia and emergency blood transfusion in Gadarif Hospital, eastern Sudan
Source: PLoS One. 2019 Dec 3;14(12):e0225731. doi: 10.1371/journal.pone.0225731 (PMC6890167; doi:10.1371/journal.pone.0225731)
Supplement: Attachment — Submitted filename: point to point 31 october.docx [file pone.0225731.s005.docx]

We would like to thank the editor and reviewers for their valuable comments on this manuscript. We greatly appreciate your time dealing with the manuscript and assessing the review comments, which enabled us to greatly improve the quality of our manuscript. Here is a point-by-point response to the reviewers’ comments and concerns

Comment

reviewer #2: All the remarks are amended, except the redundancies in the Introduction section.
When I said "redundancies", I meant: "children" two times in the first line and "mortality in children with severe anemia" two times in line 81 and 82. Please, arrange the text in a different fashion.
Figure 1 can be totally replaced by the new Table 1

Response

The redundancies in the Introduction section have been removed as suggested.

Please see the introduction now.

Figure 1 has been deleted as suggested.

Regards
